# Sequential Cryoneurolysis and Percutaneous Needle Tenotomy for Refractory Wrist Flexor Contracture: A Retrospective Case Series in Stroke and Spinal Cord Injury

**DOI:** 10.1016/j.arrct.2026.100630

**Published:** 2026-05-08

**Authors:** Paul Winston, Hendrik Pepler, Sophie Alexander, Ibrahim Ali, Mahdis Hashemi

**Affiliations:** a Faculty of Medicine, University of British Columbia, Vancouver, Canada; b Vancouver Island Health Authority, Victoria, Canada; c Canadian Advances in Neuro-orthopedics for Spasticity Consortium, Kingston, Canada; d University of Victoria, Victoria, Canada

**Keywords:** Cryoneurolysis, Muscle contracture Rehabilitation, Spasticity, Tenotomy

## Abstract

Upper limb spasticity after stroke or spinal cord injury can lead to fixed wrist flexion contractures that limit hygiene, splinting, and functional positioning. Cryoneurolysis can effectively reduce wrist flexor spastic tone; however, when longstanding contracture is present, residual mechanical restriction often persists. Percutaneous needle tenotomy (PNT) offers a minimally invasive method to release structurally shortened musculotendinous units and restore passive mobility. In this retrospective case series, we describe the outcomes of wrist flexor PNT performed after prior cryoneurolysis in patients with refractory upper limb spasticity. Four adults with chronic upper limb spasticity secondary to stroke or spinal cord injury underwent wrist flexor cryoneurolysis followed by PNT for persistent contracture limiting passive range of motion (ROM). Passive wrist extension ROM and muscle tone were graded before and after intervention. Clinical rationale for transitioning from cryoneurolysis to PNT was qualitatively documented. Across all 4 cases, cryoneurolysis produced meaningful tone reduction; however, passive wrist extension remained limited due to fixed end-range contracture. After PNT, the patients demonstrated immediate and sustained gains in passive wrist extension ROM. Modified Ashworth Scale scores remained stable or demonstrated mild improvement posttenotomy, consistent with tone reduction achieved by preceding cryoneurolysis. No major adverse events were reported. Sequential use of cryoneurolysis followed by PNT may be effective for managing refractory wrist flexion contracture, where tone reduction alone is insufficient.

## Introduction

Spasticity is a common sequela of upper motor neuron lesions, characterized by a velocity-dependent increase in muscle tone resulting from hyperexcitability of the stretch reflex arc.^1^ Spasticity can evolve into muscle stiffness, fixed postures, and joint contractures, particularly in the upper extremities, where wrist and finger flexor involvement is common, leading to impairments in function, mobility, hygiene, and quality of life. Contractures of the wrist and hand are frequently observed in patients with chronic spasticity.^2^ It has been estimated that up to 50% of individuals with moderate to severe upper limb spasticity may develop fixed flexion deformities.^3^^,^^4^

Standard management of focal spasticity typically involves physical therapies, bracing, and oral antispasmodic medications.^5^ Chemodenervation remains a cornerstone of treatment.^6^^,^^7^ However, when soft-tissue contractures are present, further interventions are required to prevent deterioration.

Cryoneurolysis is a minimally invasive treatment for focal spasticity, in which a cryoprobe is cooled to temperatures between –60°C and –88°C, is inserted percutaneously under ultrasound guidance to a targeted peripheral nerve.^8^, ^9^, ^10^ The frozen interstitial fluid forms an ice ball, inducing axonal degeneration while preserving the nerve’s epineurium and perineurium.^11^ This injury temporarily disrupts aberrant motor signaling associated with spasticity while allowing for eventual axonal regeneration.^10^^,^^11^ The efficacy of cryoneurolysis is enhanced by the use of an anesthetic diagnostic nerve block (DNB) prior to the procedure, which predicts treatment response and avoids unwanted sensory loss or muscle weakness and informs if soft-tissue contracture is present beyond the spasticity.^12^, ^13^, ^14^ Cryoneurolysis alone may not be sufficient in cases of established contracture, where biomechanical shortening of soft tissues prevents sufficient range of motion (ROM) gains even when tone is reduced. In such cases, percutaneous needle tenotomy (PNT) can be employed to release structurally shortened musculotendinous units.^15^^,^^16^

Percutaneous needle tenotomy is a minimally invasive technique in which the bevel of a large-gauge needle (typically 16-18G) is used to section or lengthen a targeted tendon through a skin puncture, performed under local anesthesia, and guided by palpation (“bowstringing”) and often ultrasound.^17^, ^18^, ^19^, ^20^, ^21^ By targeting fixed shortening of the musculotendinous unit, PNT complements tone-reducing interventions when spasticity has been mitigated, but passive ROM remains limited by contracture.^16^ Observational studies and a recent systematic review report meaningful gains in passive ROM and care goals (eg, hygiene, splinting) with low complication rates and feasibility even in frail populations.^17^, ^18^, ^19^^,^^21^^,^^22^

Both cryoneurolysis and tendon release have independently demonstrated the reduction of spasticity and improved passive ROM; however, evidence describing their sequential application is limited.^15^^,^^16^ The rationale for transitioning from cryoneurolysis to tenotomy is poorly defined. To address this gap, we evaluate wrist flexor PNT as a follow-on intervention for patients with persistent contracture despite prior cryoneurolysis. We present 4 such cases to illustrate this decision making concept. An accompanying video further illustrates the sequential approach (video S1).

## Methods

### Study design

This is a single-center retrospective case series. The ethical approval was obtained from the local ethics board (H23-00533). Each patient was informed about this study, and the proper consent form was signed by the participants. This study presents the data in accordance with the Case Report guidelines (https://www.care-statement.org/).

The pre- and postprocedure assessment was performed by independent staff who were not involved in providing the procedures to the patient to minimize observer bias. Demographic information, wrist extension, active range of motion (AROM), and the maximal passive ROM (known as V1) were measured based on the Modified Tardieu scale.^23^ The wrist flexor muscle spasticity was measured based on the Modified Ashworth Scale (MAS).^24^

### Cryoneurolysis

Diagnostic nerve blocks were first performed under ultrasound guidance with 2% lidocaine and electrical stimulation, targeting a combination of the intramuscular median nerve branches to flexor carpi radialis (FCR), palmaris longus (PL), flexor digitorum superficialis (FDS), and ulnar nerve branches to flexor carpi ulnaris (FCU), in accordance with previously published protocols.^12^ Post-DNB assessments were conducted to ensure safety and effectiveness. Significant improvements in V1, AROM, and MAS score of the wrist placed into extension, after the DNB, were considered indicative of potential benefit from cryoneurolysis.

The cryoneurolysis to the targeted muscles was performed in accordance with published guidelines.^8^^,^^25^ Cryoneurolysis was delivered with the *Iovera* handheld device (Iovera System 190 Smart Tip; Iovera, Pacira).^a^

### Percutaneous needle tenotomy

Under ultrasound guidance, the forearm and the region over the targeted wrist flexor tendons were prepared with an antiseptic technique. Doppler flow was used to avoid blood vessels. After palpation to identify the tendons, 1-3 mL of 2% lidocaine was infiltrated around the tendons under ultrasound visualization. A 16-gauge Admix Nokor needle (BD Angiocath TM Becton, Dickinson and Company)^b^ was then introduced into the tendon, and gentle longitudinal movements were performed to release the tendon. This process was continued until a satisfactory release was achieved, as confirmed by an improved ROM.^22^

### Illustrative cases

#### Case 1

A 54-year-old female with a history of pontine stroke related to vertebrobasilar arterial dissection occurring in 2010 presented to this clinic regarding spasticity in the left upper and lower extremities.

After a successful DNB to multiple targets, cryoneurolysis was performed on January 19, 2024, targeting the elbow flexors and pronator teres, FCR, and flexor digitorum superficialis (FDS).

Despite measurable gains in ROM and reduction in tone after cryoneurolysis, contracture remained in the wrist, and PNT was pursued due to residual limitation that appeared driven by fixed soft-tissue restriction rather than ongoing reversible spasticity. Of note, the patient also had a prior PNT to the biceps brachii tendon^22^ with good results and was eager to proceed with the wrist. The PNT began with the FCR (she does not have a palmaris longus) until the wrist could relax into full extension, followed by additional proximal release of the FDS due to persistent finger flexor tightness, which further improved ROM; there was minimal bleeding and no swelling or bruising. Full passive range of motion (V1) occurred with a reduction in the MAS seen at the 1-year follow-up (table 1).

**Table 1:** Patient 1 wrist extension measurements postcryoneurolysis and FCR and FDS tenotomy

	Date	Days Since Procedure	V1	V3	AROM	MAS
Cryo 1 Nerves treated: FCR, pronator teres, biceps, brachioradialis	Jan 19, 2024	Baseline	75	No	No	3
	Feb 20, 2024	32	90	45	No	1+
	June 14, 2024	147	80	−20	No	2
	Jan 14, 2025	361	70	10	No	2
Wrist tenotomy tendons treated: FCR and FDS	Jan 14, 2025	Baseline	70	10	No	2
	Aug 1, 2025	199	85	10	No	2
	Dec 10, 2025	330	90	−5	No	2/3

Abbreviations: AROM, active range of motion; FCR, flexor carpi radialis; FDS, flexor digitorum superficialis; MAS, Modified Ashworth Scale.

#### Case 2

A 71-year-old female with right side hemiparesis and spasticity due to left hemorrhagic basal ganglion stroke in 2016, who was referred for management of bothersome spasticity with concern for evolving contracture formation, including impaired wrist and hand opening and reduced forearm supination. After a successful DNB, she proceeded with 3 cryoneurolysis sessions. The first cryoneurolysis on February 14, 2023, targeted the shoulder and elbow muscles and the lower leg, as well as the median nerve above the elbow. Her gains were sufficient to have active opening of the wrist and fingers from a nonfunctional hand. The patient chose to have 2 further cryoneurolysis of the upper extremity as she found it relieved her symptoms and allowed active hand opening, at 9 months and 11 months apart. Despite ROM improving after cryoneurolysis, the wrist remained tight, and at follow-up, it was concluded that percutaneous lengthening of the FCR for contracture was warranted; PNT was performed on 95 days after the prior cryoneurolysis to the FCR and palmaris longus with no adverse events.

Before the scheduled posttenotomy follow-up, the patient suffered a fall on her right outstretched arm, suffering a displaced fracture of the right humeral head. Despite extensive pain in the right wrist, no fractures were detected on CT. Cryoneurolysis was performed on the suprascapular nerve for shoulder pain only. Seventeen months post-PNT, she had full passive ROM at the wrist (table 2).

**Table 2:** Patient 2 wrist extension measurements for cryoneurolysis procedures and FCR and PL tenotomy

	Date	Days Since Procedure	V1	V3	AROM	MAS
Cryo 1 Median nerve above the elbow	Feb, 2023	Baseline	15	−10	−15	3
	May, 2023	77 d	65	No	15	2
	Sept, 2023	164 d	60	No	20	2
	Oct, 2023	253 d	30	20	5	3
Cryo 2 Nerves treated: Palmaris longus, FCU, FCR, and FDL		Baseline	30	20	5	3
	Nov, 2023	26 d	50	15	10	2
	Jan, 2024	90 d	50	20	−15	2
	July, 2024	261 d	30	−35	No	3
Cryo 3 Nerve treated: FCU		Baseline	30	−35	No	3
	Oct, 2024	34 d	40	−45	−25	3
Wrist tenotomy Tendons treated: FCR and Palmaris Longus	Oct, 2024	Baseline	40	−45	−25	3
	March, 2026	525 d	70	−5	10	2/3

Abbreviations: AROM, active range of motion; FCR, flexor carpi radialis; FCU, flexor carpi ulnaris; FDL, flexor digitorum longus; MAS, Modified Ashworth Scale; PL, palmaris longus.

#### Case 3

A 46-year-old female with a history of cervical spinal cord injury (C3 ASIA D) and had bilateral upper-extremity spasticity and progressive stiffness affecting shoulder positioning and wrist extension, raising concern for evolving fixed contracture. She had previously undergone cryoneurolysis to the pectoral region with symptomatic benefit, and repeat cryoneurolysis was pursued to address recurrent tone and pain in the wrist.

On October 18, 2024, she underwent ultrasound-guided cryoneurolysis targeting the shoulder muscles for shoulder spasticity and pain control, as well as cryoneurolysis to the PT, FCR, FCU, and FDS. Despite improved shoulder comfort and wrist ROM, distal wrist stiffness persisted, and examination suggested a fixed soft-tissue restriction rather than ongoing reversible spasticity, prompting PNT.

She underwent wrist PNT on January 3, 2025. The release of the FCR alone resulted in immediate improvement in wrist extension. At 102 days post-PNT, wrist ROM and tone had improved, and at 193 days post-PNT, improvements were largely sustained confirming a durable mechanical benefit from PNT after maximal achievable tone reduction. At approximately 2 months post-PNT, the patient reported a localized bulge and discomfort at the procedure site, exacerbated by weight bearing. The examination identified a 1 × 2 cm firm lump, and ultrasound demonstrated a small fluid collection around the tendon sheath, which was managed *y* with continued physiotherapy and range-of-motion exercises. At 3 months posttenotomy, persistent localized pain and swelling prompted treatment with a 20-mg Depo-Medrol injection combined with 1 cc of lidocaine, resulting in improved comfort and range of motion (table 3).

**Table 3:** Summary of patient 3 wrist extension measurements postcryoneurolysis and FCR tenotomy

	Date	Days Since Procedure	V1	V3	AROM	MAS
Cryo 1 Nerves targeted: pronator teres, FCR, FDS, FCU	Oct 18, 2024	Baseline	45	No	25	2/3
	Nov, 2024	25 d	60	20	10	2
	Jan, 2025	77 d	60	20	15	3
Wrist tenotomy Tendon treated: FCR		Baseline	60	20	15	3
	April, 2025	102 d	65	−10	25	1+
	July, 2025	193 d	70	25	20	1
	March, 2026	438 d	70	30	30	1`

Abbreviations: AROM, active range of motion; FCR, flexor carpi radialis; FCU, flexor carpi ulnaris; FDS, flexor digitorum superficialis; MAS, Modified Ashworth Scale.

#### Case 4

A 70-year-old male with Parkinson’s disease and a hemorrhagic stroke resulting in left-hemiparetic spasticity was referred for subacute poststroke left upper limb complex regional pain syndrome due to pain, swelling, and stiffness, with concern for contracture formation that limited comfort, mobility, and functional use. The patient was treated with oral prednisone for complex regional pain syndrome, resulting in significant improvement in pain and swelling and reduced stiffness at the wrist, fingers, and shoulder at 3-week follow-up. This unmasked his spasticity, and botulinum toxin injections were subsequently administered to the PT, FCR, and FCU, with partial improvement in hand pain and stiffness observed at 3 months and repeated. After the 2 treatments, the patient desired a longer-term treatment strategy, cryoneurolysis targeting the shoulder musculature and the median nerve above the elbow, which was offered in October 2022.^25^

Although these gains were maintained, persistent wrist spasticity prompted repeat cryoneurolysis targeting the wrist flexor muscles—including FCR, FDS, and PT in 2024. This provided a meaningful reduction in tone and pain and helped him achieve improved ROM; he continued to have significant stiffness and refractory contracture, with the physical examination noting palpable FCR and PL tendons and persistent limitation in dexterity and sustained end-range positioning, likely compounded by Parkinsonian rigidity. A wrist flexor PNT was subsequently performed in March 2025 to the PL and FCR (table 1). The PNT was well tolerated with minimal bleeding controlled with a small bandage and minimal pain, and the recorded outcome was marked improvement including full passive ROM, with the only persisting issue being a palpable tendon band that did not bother him, although he noted the arm could feel heavy when running. He had ongoing improvements in the rest on follow-up until 168 days. The range began to decrease 264 days after the PNT, but further treatment was not required (table 4).

**Table 4:** Patient 4 wrist extension measurements postcryoneurolysis and FCR and PL tenotomy

	Date	Days Since Procedure	V1	V3	AROM	MAS
Cryo 1 Nerves treated: Median nerve above the elbow	Oct, 2022	Baseline	50°	No	30°	3
	Dec, 2022	52 d	65°	50°	30°	2
	April, 2023	182 d	65°	40°	30°	1
	August, 2023	287 d	70°	No	45°	1+
	Jan, 2024	462 d	70°	No	50°	1+
Cryo 2 Nerves treated: left FCR, pronator teres, FDS, and FCU	Jan, 2024	Baseline	70°	No	50°	1+
	April, 2024	69 d	80°	70°	55°	1+
	July, 2024	146 d	75°	45°	55°	3
	Nov, 2024	237 d	80°	40°	50°	1+
Cryo 3 Nerves treated: left FCR, pronator teres, FDS, and FCU	Nov, 2024	Baseline	80°	40°	50°	1+
Wrist flexor tenotomy Tendons treated: FCR and Palmaris Longus	March, 2025	Baseline	65°	20°	20°	2
	May, 2025	60 d	85°	10°	30°	1+
	August, 2025	168 d	85°	50°	60°	1+
	Nov, 2025	264 d	75°	40°	40°	2

Abbreviations: AROM, active range of motion; FCR, flexor carpi radialis; FCU, flexor carpi ulnaris; FDS, flexor digitorum superficialis; MAS, Modified Ashworth Scale; PL, palmaris longus.

## Discussion

To our knowledge, this is the first study to examine the sequential use of cryoneurolysis followed by PNT for refractory wrist flexor contracture. The patients were followed for long periods. In this case series, cryoneurolysis reduced wrist flexor tone but did not fully restore passive wrist extension instead unmasking a firm, velocity-independent end-range limitation with palpable tendon restriction indicative of a mechanical rather than neural barrier to further ROM gains. Escalation to PNT was supported by a lack of tone reduction after DNB and cryoneurolysis, persistence of a firm end-range block, evidence of tendon shortening with palpable restriction, and functional goals centered on positioning or hygiene. Percutaneous needle tenotomy produced immediate and durable improvements in passive wrist extension and ongoing stable or improved MAS.

Prior studies of cryoneurolysis have demonstrated improvements in tone, pain, and range of motion across multiple upper limb targets, including the wrist, with effects lasting up to 1 year in some cohorts.^8^^,^^16^ However, ROM beyond the improvement of a nerve-targeted procedure is required. The PNT thus served as a targeted intervention for the mechanical component of impairment revealed after maximal tone modulation. The irreversibility of PNT underscores the value of a staged approach, in which neural contributors are addressed first, and contracture release is reserved when fixed tissue restriction is clearly established. This sequencing allows clinicians to avoid premature PNT while still achieving meaningful gains in passive ROM when conservative and neuroablative strategies alone are insufficient.

The sequential strategy described here may offer a framework for managing complex upper limb spasticity, particularly in patients who have plateaued. Rather than viewing tone reduction and contracture release as competing approaches, our findings support their integration into a mechanism-driven, staged treatment pathway. This framework aligns with a growing emphasis on individualized spasticity management.

This cohort represents cases of severe spasticity, with little functional movement. Contracture of the wrist is more common in those with limited or absent active movements.^2^ Over time, muscles can continue to undergo rheologic changes, due to the persistent muscle properties of spastic muscles and paralysis.^3^^,^^4^

The PNT is an inexpensive procedure, requiring only a small needle and injectable anesthetic. It is an adjunct to the cyroneurolysis, which has been found to be cost-efficient compared with other injection-based treatments for spasticity, such as botulinum toxin.^26^

One patient noted a nonpainful lump at the site of PNT, and another had pain, which was managed locally. This will inform future techniques. The painful lump was located too distally in the wrist.

### Limitations

The retrospective design of 4 cases limits generalizability and precludes statistical analysis. Outcome measures were restricted to routinely collected clinical data, including ROM and MAS scores in nonfunctional hands and fingers, making testing of power and function difficult. Additionally, follow-up duration varied across cases. Prospective studies with larger cohorts, standardized outcome measures, and specified follow-up are needed to validate these findings and refine criteria for treatment sequencing.

## Conclusions

This retrospective case series suggests that sequential use of cryoneurolysis followed by PNT may be an effective strategy for managing refractory wrist flexor contracture in patients with chronic upper limb spasticity. Cryoneurolysis can provide durable tone reduction and serves as a critical diagnostic step in distinguishing reversible spasticity from fixed soft-tissue contracture. When the passive ROM remains limited despite adequate tone control, PNT may improve mechanical mobility and achieve sustained improvements in joint positioning. These findings support a stepwise, mechanism-driven approach to spasticity management.

## Suppliers

a. *Iovera* handheld device; Iovera System 190 Smart Tip, Iovera, Pacira.
b. 16-gauge Admix Nokor needle; BD Angiocath TM Becton, Dickinson and Company.

## Disclosures

P.W. has received honoraria for speaking engagements and has been on the Ad Board for Pacira. He has received funding for investigator-initiated trials funded by Pacira via his institution.

## Appendix Supplementary materials

Video. Wrist tenotomy cases after cryoneurolysis Image, video 1 dummy alt text
